# Preliminary histopathological study of intra-articular injection of a novel highly cross-linked hyaluronic acid in a rabbit model of knee osteoarthritis

**DOI:** 10.1007/s10735-012-9457-4

**Published:** 2013-02-07

**Authors:** Tommaso Iannitti, Mohamed Elhensheri, Ali Ö. Bingöl, Beniamino Palmieri

**Affiliations:** 1 Department of Physiology, School of Medicine, University of Kentucky Medical Center, 800 Rose Street, Lexington, KY 40536-0298 USA; 2 Department of Experimental Pathology, Bioserv Analytic and Medical Devices GmbH, Rostock, Germany; 3 Department of Chemistry, University of Hamburg, Hamburg, Germany; 4 Department of General Surgery and Surgical Specialties, University of Modena and Reggio Emilia Medical School, Surgical Clinic, Modena, Italy

**Keywords:** Osteoarthritis, Hyaluronic acid, Intra-articular, Injection, Corticosteroid, Collagenase, Variofill^®^, Knee

## Abstract

Osteoarthritis is a degenerative joint disease mostly occurring in the knee and commonly seen in middle-aged and elderly adults. Intra-articular injection of hyaluronic acid has been widely used for treatment of knee osteoarthritis. The aim of this study was to evaluate the efficacy of intra-articular injection of a novel highly cross-linked hyaluronic acid, alone or in combination with ropivacaine hydrochloride and triamcinolone acetonide, on knee articular cartilage in a rabbit model of collagenase-induced knee osteoarthritis. After induction of experimental osteoarthritis by intra-articular injection of collagenase, adult New Zealand white rabbits (n = 12) were divided into 3 groups. Group 1 (control group) received 0.3 ml phosphate buffered saline into the right knee joint. Group 2 received 0.3 ml cross-linked hyaluronic acid (33 mg/ml) into the right knee joint. Group 3 received a mixture of 0.15 ml cross-linked hyaluronic acid (33 mg/ml), 0.05 ml ropivacaine hydrochloride 1 % and 0.1 ml triamcinolone acetonide (10 mg/ml) into the right knee joint. Intra-articular injections were given 4 weeks after first collagenase injection and were administered once a week for 3 weeks. Gross pathology and histological evaluation of rabbits’ knee joints were performed after 16 weeks following initial collagenase injection. Histological analysis of sections of right knee joints at lesion sites showed a significant decrease in Mankin’s score in groups treated with hyaluronic acid alone or in combination with ropivacaine hydrochloride and triamcinolone acetonide versus control group (*p* < 0.05 and *p* < 0.01 respectively). This evidence was consistent with strong articular degenerative changes in control right knee joints (grade III osteoarthritis), while the treated groups revealed less severe articular degenerative changes (grade II osteoarthritis). The present results show that cross-linked hyaluronic acid, alone or in combination with ropivacaine hydrochloride and triamcinolone acetonide, produces a significant improvement in knee articular cartilage degeneration in a rabbit model of collagenase-induced osteoarthritis.

## Introduction

Osteoarthritis (OA) is a degenerative joint disease mostly occurring in the knee and commonly seen in middle-aged and elderly adults. Knee OA is one of the major causes of pain and disability. OA-related pain and disability significantly affect the patients’ quality of life. Non-surgical procedures to manage OA include weight loss, exercise, activity modification, assistive devices, non-steroidal anti-inflammatory drugs (NSAIDs), analgesics, and intra-articular (IA) hyaluronic acid (HA) injection (Hochberg et al. 1995; Iannitti et al. 2011). Current symptom management is directed to relieve pain regaining function (Elron-Gross et al. 2008). However, treatments available are limited and outcomes are poor due to the high incidence of side effects. For example, oral administration of NSAID tablets relieves inflammation and pain, but may cause serious gastrointestinal adverse effects. IA drug delivery can be useful in order to treat joint inflammation and pain. IA HA injection is a well-documented treatment for knee OA and is approved worldwide (Jiang et al. 2010; Gigante and Callegari 2011). HA is a glycosaminoglycan responsible for the maintenance of healthy synovial fluid viscoelasticity (Bingöl et al. 2010). These peculiar properties of HA guarantee an extremely low-friction environment within the joints. Arthropathies lead to a reduction in synovial fluid viscoelasticity due to a decrease in concentration and/or HA molecular weight (Altmann et al. 1980). A high synovial fluid viscoelasticity is necessary for lubrication, shock absorption and load-bearing and is required to prevent mechanical trauma and articular cartilage wear during load-bearing movement (Schurz 1996). IA HA injection aims at decreasing OA-related pain and improving joint function by restoring synovial fluid physiological properties (Balazs and Gibbs 1970). There has been a considerable controversy about whether HA therapy has structure-modifying properties. IA HA administration produces well documented structure-modifying effects, as observed in several animal species. For instance, Wiig et al. (1990) reported that administration of a single injection of Healon, immediately after anterior cruciate ligament transaction in rabbits, resulted in significantly decreased inflammation, increased collagen synthesis and angiogenesis and enhanced tissue repair if compared to a single injection of saline solution (Wiig et al. 1990). Furthermore, Armstrong et al. (1994) and Kikuchi et al. (1996) investigated the structure-modifying effects of HA using a sheep and rabbit model of meniscectomy (Armstrong et al. 1994; Kikuchi et al. 1996). IA injection of HA, after partial meniscectomy in these species, resulted in significantly inhibited cartilage degeneration (Armstrong et al. 1994; Kikuchi et al. 1996). A further study by Marshall et al. (2000) evaluated Synvisc in a dog anterior cruciate ligament transaction model (Marshall et al. 2000). Gross morphological and histological damage within joints that received injections were significantly milder if compared to control joints (Marshall et al. 2000). The positive effect of IA HA in patients affected by knee OA has also been observed in many clinical studies. For example, Leardini et al. (1991) studied 40 patients affected by this condition and reported that the IA injection of HA was as effective as cortisone, and the side effects of both treatments were of short duration and negligible. Another study compared HA and cortisone administration in 63 patients with knee OA once a week for 5 weeks (Jones et al. 1995). This study showed that pain was less severe in the HA group if compared with the cortisone group after a 6-month follow up. Pietrogrande and coworkers compared the activity and tolerability of HA and cortisone administration in patients affected by knee OA (Pietrogrande et al. 1991). HA was administered once a week for 5 weeks and cortisone once a week for 3 weeks. They observed that the latter had a more rapid action (Pietrogrande et al. 1991). Song and Chang reported that IA injections with either HA or triamcinolone acetonide are effective for treatment of knee OA with no significant differences in the outcome (Song and Chang 2004). On the other hand, a study in the rabbit showed that, in the long term, HA was more effective than cortisone as far as degenerative knee OA healing was concerned (Karakok et al. 2001). Furthermore, a combination of cortisone and HA was the most effective in treatment of cartilage degeneration in a rabbit model of knee OA, if compared to HA alone (Karakurum et al. 2003). IA HA also appears to have a long-lasting effect in relieving knee OA-related pain if compared to IA corticosteroids. This was pointed out in a meta-analysis by Bannuru et al. (2009) where, from baseline to week 4, IA corticosteroids were relatively more effective for pain relief if compared to IA HA but, by week 4, the 2 approaches had equal efficacy and, beyond week 8, HA had greater efficacy. This trend is of particular importance for clinicians using IA HA for treatment of knee OA.

Recently, local drug delivery has been widely used for treatment of OA. Drug delivery strategies may reduce local joint inflammation and joint destruction, offering pain relief, and restoring the patient’s activity levels and joint function (Allen et al. 2010). One of the most important advantages of IA drug administration is that it can achieve a targeted delivery of the drug to affected tissues allowing local treatment and minimizing side effects typical of systemically administered drugs (Edwards et al. 2007). In this context micro- and nanocarrier-mediated drug delivery systems, including polymeric particles and hydrogel, are well-established as methods for sustained release of drugs into the IA space (Zhang and Huang 2012). Drug delivery systems could prolong drug retention time, reduce the drug clearance into the joint cavity, and increase the patient’s compliance as well as the therapeutic effect of pharmaceutical agents. IA HA injection, alone or in combination with other drugs, is also widely used in the clinic with a frequency and dosage calculated on the basis of the disease pathophysiology (Iannitti et al. 2011). The previously described studies show that the scientific literature has widely reported on IA HA/cortisone injection for treatment of knee OA, sometimes with controversial results. However, while in many animal studies the efficacy of non-cross-linked HA solution alone or combined with other drugs has been analyzed, evidence of the efficacy of cross-linked HA gel combined with other pharmaceutical products is still lacking. The hydrogel form is characterized by cross-linked HA, which allows a longer permanence within the body, and it is also characterized by greater viscoelastic properties if compared with the non-cross-linked form of HA (Oh et al. 2008; Shimojo et al. 2010).

## Aim

The aim of the present study was to evaluate the efficacy of IA injection of a novel cross-linked HA, alone or in combination with ropivacaine hydrochloride and triamcinolone acetonide, on knee articular cartilage in a rabbit model of collagenase-induced knee OA.

## Materials and methods

### Animals

Twelve female New Zealand white rabbits (age = 12 weeks; weight: 4080–5320 g) were used in this study. The animals were kept at a temperature of 21–23 °C and a relative humidity of 40–60 %. The animals were allowed free access to water and they were fed on a rabbit commercial diet. Animal experiments were performed in accordance with document DIN EN ISO 10993-2 and according to Germany Animal Welfare Act (Lorz 1987).

### Preparation of collagenase solution

Collagenase type II from *Clostridium histolyticum* (Sigma-Aldrich, Germany) was dissolved in a sterile phosphate buffered saline (PBS; Sigma-Aldrich, Germany) solution (400 U/ml, pH 7.4) and filtered with a 0.22 μm membrane.

### Animal model of knee osteoarthritis

Animals were anesthetized with a subcutaneous injection of Ketamin (0.15 ml/kg Ketamin 10 %, Ceva Tiergesundheit GmbH, Düsseldorf, Germany) and Medetomidin (0.1 ml/kg Domitor, Pfizer GmbH, Karlsruhe, Germany). Rabbit knee joints were shaved and sterilized, and 0.1 ml collagenase solution was slowly injected into the right knee joint cavity. The same collagenase injection procedure was performed 3 days after the first injection to develop experimental OA according to Kikuchi et al. (1998).

### Cross-linked hyaluronic acid

Variofill^®^ (33 mg/ml, Adoderm, Langenfeld, Germany) is a novel divinyl sulfone cross-linked HA engineered to form a highly cross-linked gel with high viscoelastic properties.

### Pharmacological treatment

Animals were divided into 3 groups: (1) 4 rabbits were used as control group and received 3 injections (day 0, 7, 14) of 0.3 ml PBS into the right knee joint; (2) 4 rabbits received 3 injections (day 0, 7, 14) of 0.3 ml HA (Variofill^®^; 33 mg/ml) into the right knee joint; (3) 4 rabbits received 3 injections (day 0, 7, 14) of 0.15 ml HA (Variofill^®^; 33 mg/ml) plus 0.05 ml ropivacaine hydrochloride 1 % (Naropin^®^, AstraZeneca, Wedel, Germany) plus 0.1 ml triamcinolone acetonide (Valon^®^ A10, Dermapharm AG, Grünwald, Germany; 10 mg/ml) [mixed before the injection] into the right knee joint.

### Preparation of hyaluronic acid plus ropivacaine hydrochloride and triamcinolone acetonide mixture

In order to create a mixture of ropivacaine hydrochloride, triamcinolone acetonide and HA, a 1-ml syringe, containing 0.05 ml of ropivacaine hydrochloride 1 % plus 0.1 ml of triamcinolone acetonide (10 mg/ml), was attached to a syringe containing 0.15 ml of HA (33 mg/ml) through a Rapid Fill Connector Luer Lock-to-Luer Lock (Baxa, Englewood, CO). A continuous push-pull action between syringes was performed in order to create a homogeneous mixture (optimal results require 20 “passes” between syringes).

### Gross pathological observation and histological evaluation

After 16 weeks from the initial collagenase injection, rabbits were sacrificed using intravenous injection of ketamine (0.15 ml/kg) followed by 0.3 ml/kg intravenous injection of T61 (Intervet Deutschland GmbH, Unterschleißheim, Germany) for gross pathological observation and histological evaluation. Rabbits’ knee joints were dissected after euthanasia. Knee joint tissue was fixed in 10 % neutral-buffered formalin, decalcified in EDTA for 2 weeks and 3 µm sections of the central portion of the knee joint were stained with Hematoxylin and Eosin (HE) and Safranin-Orange-fast green (Safranin-O) stainings. Safranin-O staining was used for the evaluation of degenerative cartilage lesions. Histological samples were graded using a modified Mankin’s score (0–2: normal; 3–5: OA grade I; 6–7: OA grade II; 8–10: OA grade III; 11–14: OA grade IV) to evaluate structure, fissures, clefts, cells, vascularity and saturation of articular cartilage (Kuroki et al. 2004). The maximum score can be achieved when facing heavily disintegrated and degenerated cartilage.

### Statistical analysis

Mankin’s score data were first checked for normality by means of the Anderson–Darling test using Minitab, v15. Statistical analysis was performed using GraphPad Prism 5 software. A one-way ANOVA, followed by Bonferroni post hoc test, was used to compare treatment groups. A value of *p* < 0.05 was considered significant. Data are presented as the mean ± Standard Error of the Mean (SEM).

## Results

### Gross pathological analysis

**Fig. 1:**
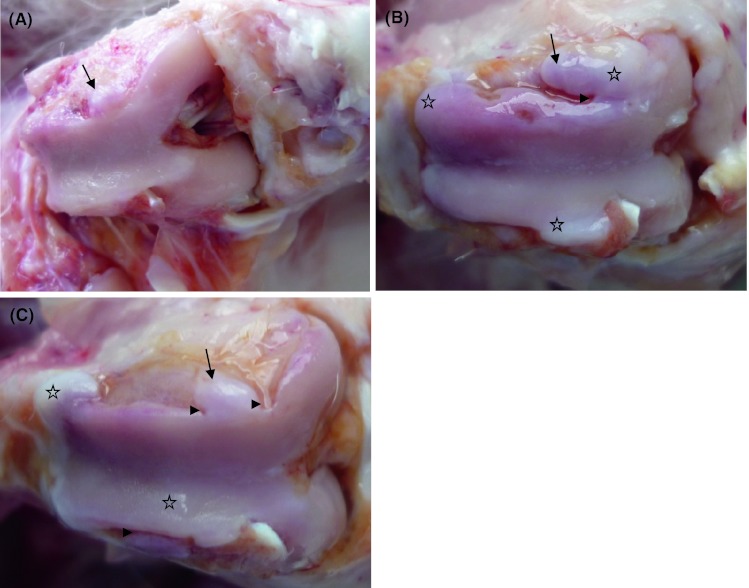
**A** Right knee joint of control group rabbit: matt, unpolished and irregular articular surface with osteophyte-like structure (*arrow*) at the marginal zone; **B** Right knee joint of HA group rabbit: matt, unpolished and irregular articular surface with osteophyte-like structure (*arrow*), different grooves (*arrowhead*) and newly white fibrocartilaginous or cartilage-like tissue (*stars*) on the marginal zone; **C** Right knee joint of HA plus ropivacaine hydrochloride and triamcinolone acetonide group rabbit: matt, unpolished and irregular articular surface with osteophyte-like structure (*arrow*), different grooves (*arrowheads*) and newly white fibrocartilaginous or cartilage-like tissue (*stars*) on the articular surface

No changes typical of degeneration were observed in the left joint of treatment and control group animals. Left knee joint articular cartilage was characterized by a smooth, polished and regular surface in treatment and control group animals. Articular cartilage in right knee joint presented with a matt, unpolished and irregular surface (Fig. 1A, B, C). In both treatment groups, cartilage lesions, associated with OA, were clearly seen, but the severity was milder than in the control group. Here, in the marginal zone of trochlea ossis femoris, condylus lateralis femoris and condylus medialis femoris, osteophyte-like structures and different grooves were observed.

### Histopathological findings

#### Group 1

**Fig. 2:**
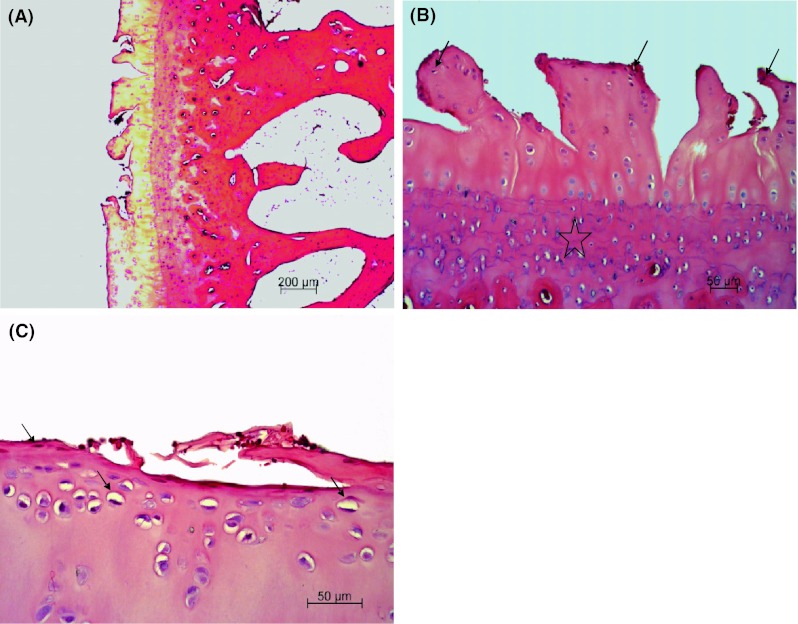
**A** Photomicrograph of sagittal section of condylus femoris of the right knee joint of control group rabbit showing focal erosion including fibrillation and multifocal vertical fissures extending into the deep zone of articular cartilage. HE staining; **B** Higher magnification of panel **A** showing articular cartilage erosion, tidemark destruction (*star*) and chondrocyte degeneration (*arrows*). HE staining; **C** Transverse section of trochlea ossis femoris of the right knee joint of control group rabbit showing focal erosion including fibrillation, superficial peeling and chondrocyte degeneration (*arrow*) of articular cartilage. HE staining

**Fig. 3:**
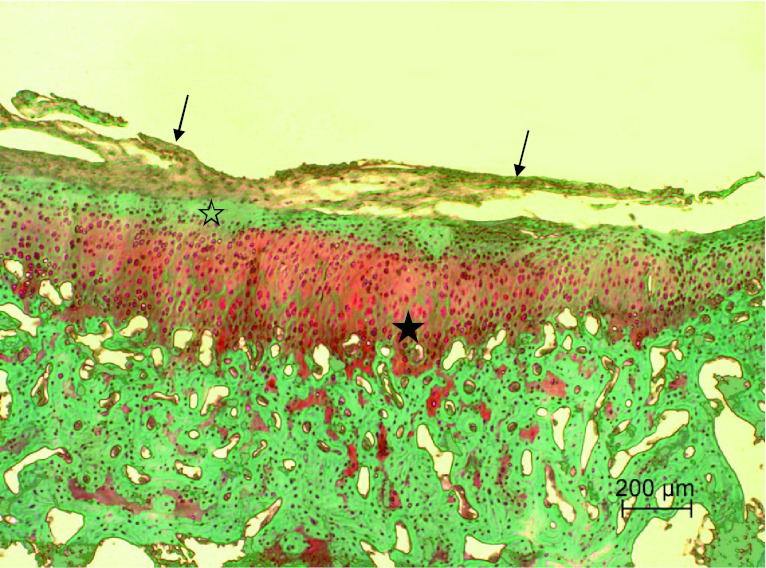
Articular surface of the right knee joint of control group rabbit showing fibrocartilage tissue (*arrows*) covering injured articular surface with moderate lack of Safranin-O staining in superficial zone (*open star*) and irregular staining distribution (depletion of proteoglycans; *star*) in middle and deep zones. Safranin-O staining

**Fig. 4:**
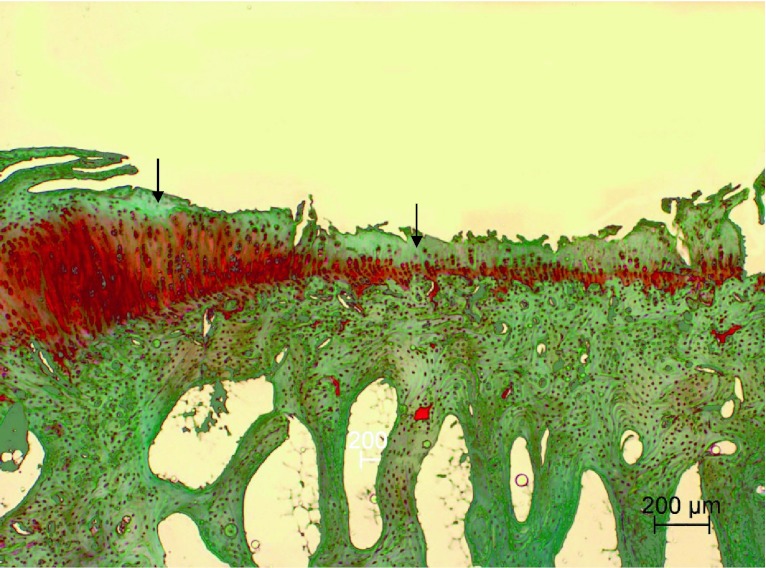
Articular surface of the right knee joint of control group rabbit showing severe erosion extended to the deep zone with moderate lack of Safranin-O staining (*arrows*) and irregular staining distribution (depletion of proteoglycans) in middle and deep zones of the erosion area. Safranin-O staining

In the right knee joint, all animals revealed superficial fibrillation of articular cartilage, minimal chondrocyte clustering and fibrosis in the superficial zone, multifocal extension of matrix cracks into the mid zone to form vertical fissures (clefts), destroyed tidemark structures and proliferation of fibrous tissue including active fibroblasts with prominent nuclei and abundant cytoplasm present at the margin of the trochlea ossis femoris, condylus lateralis femoris, condylus medialis femoris and tibial plateau (Fig. 2A, B, C). Additionally, some adjacent trabeculae, along the junction between damaged cartilage and subchondral bone, were lined by osteoblasts present in a rabbit. Multifocal extensive erosion of superficial zone of condylus femoris was observed in two animals. The hyaline cartilage showed moderate reduction in matrix coloration, which was more extensive in superficial and cracked areas, as assessed by Safranin-O staining (Figs. 3, 4). The right knee synovium showed moderate hypertrophy/thickening of synovial lining cell layers, multiple finger-like projections, villi formation and moderate hypertrophy of subsynovial tissue with few inflammatory infiltrates and granulation tissue formation including multiple angiogenesis. Mankin′s score in the control group was 10 ± 0.4 points, which corresponds to grade III OA.

#### Group 2

**Fig. 5:**
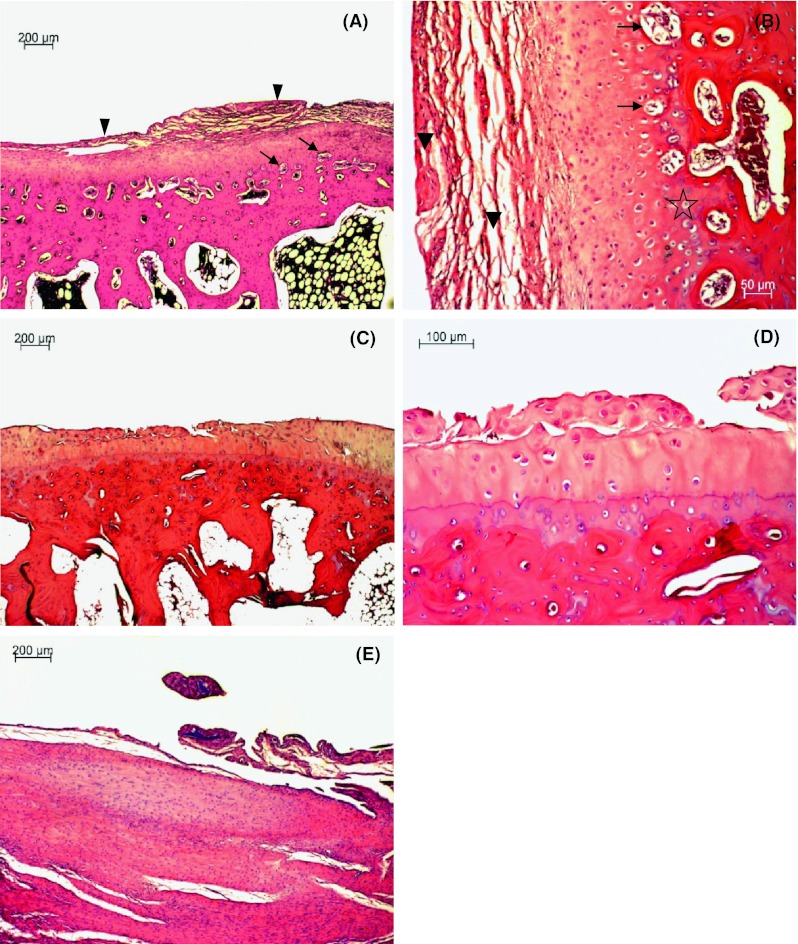
**A** Photomicrograph of transverse section of trochlea ossis femoris of the right knee joint of HA group rabbit showing fibrocartilage tissue covering injured articular surface (*arrowheads*) and multiple angiogenesis (*arrows*) of articular cartilage. HE staining; **B** Higher magnification of panel **A** showing fibrocartilage tissue covering injured articular surface (*arrowheads*), multiple angiogenesis (*arrows*) and tidemark destruction (*star*) of articular cartilage. HE staining; **C** Sagittal section of condylus femoris of the right knee joint of HA plus ropivacaine hydrochloride and triamcinolone acetonide group rabbit showing focal erosion of articular cartilage with chondrocyte hyperplasia and new cartilage tissue formation. HE staining; **D** Higher magnification of panel **C** showing focal erosion of articular cartilage with chondrocyte hyperplasia and new cartilage tissue formation. HE staining; **E** Synovium of the right knee joint of HA group rabbit showing hypertrophy (thickening) of synovial lining cell layers, oligofocal pieces of basophilic foreign material surrounded by granulation tissue including foreign-body giant cells, lymphocytes and fibrosis, hypertrophy of subsynovial tissue with few inflammatory infiltrates and multiple angiogenesis. HE staining

**Fig. 6:**
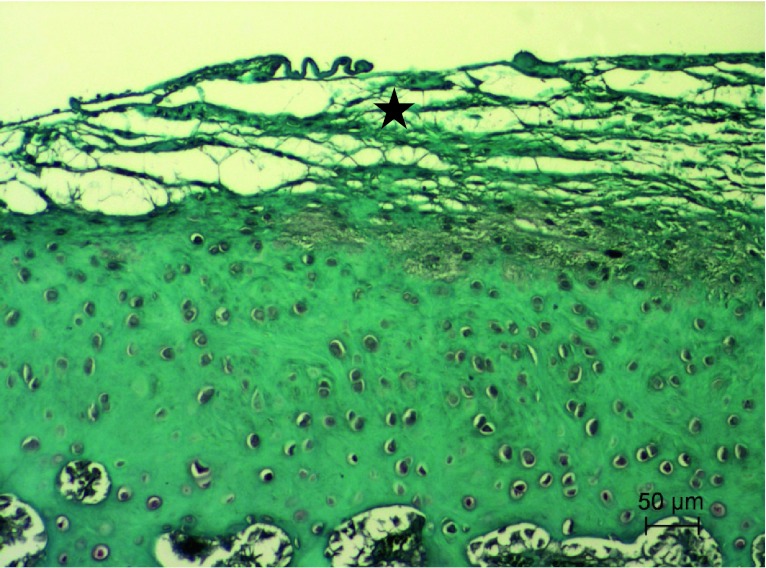
Articular surface of the right knee joint of HA group rabbit showing fibrocartilage tissue (*star*) covering injured articular surface with severe lack of Safranin-O staining in hyaline cartilage matrix. Safranin-O staining

In 3 rabbits, articular cartilage of the right knee joint contained multiple chondrocytes and disoriented chondrocytes became more prominent. The right knee synovium revealed moderate hypertrophy (thickening) of synovial lining cell layers punctuated by oligofocal pieces of basophilic foreign material, multiple finger-like projections, villi formation, and moderate hypertrophy of subsynovial tissue with few inflammatory infiltrates and granulation tissue formation including multiple angiogenesis (Figs. 5A, B, E, 6). A rabbit died in the initial phase of the study and was excluded from the modified Mankin’s score evaluation. Mankin′s score in the HA treated group was 7.3 ± 0.6 points, which corresponds to grade II OA. Therefore a significant decrease in Mankin’s score was observed in this group versus group 1 (*p* < 0.05).

#### Group 3

**Fig. 7:**
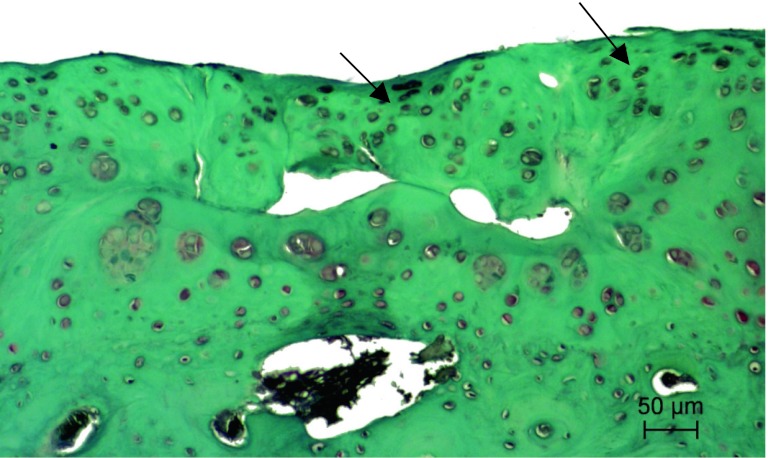
Articular surface of the right knee joint of HA plus ropivacaine hydrochloride and triamcinolone acetonide group rabbit showing focal erosion of articular cartilage and chondrocyte proliferation (*arrows*) with severe lack of Safranin-O staining in new cartilage formation and hyaline cartilage matrix. Safranin-O staining

**Fig. 8:**
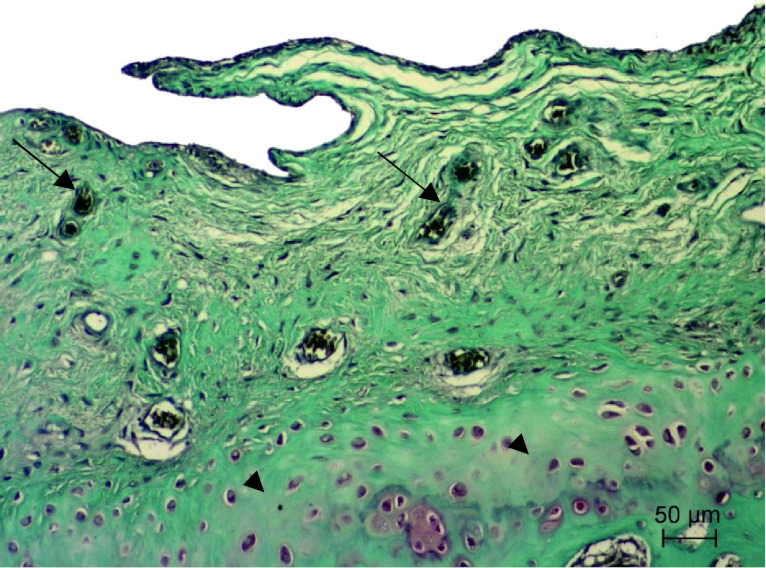
Transverse section of trochlea ossis femoris of the right knee joint of HA plus ropivacaine hydrochloride and triamcinolone acetonide group rabbit showing multiple angiogenesis (*arrows*) in the newly formed fibrocartilage tissue with severe lack of Safranin-O staining in articular cartilage matrix (*arrowheads*). Safranin-O staining

Changes in articular cartilage and synovium, including modified Mankin’s score evaluation of the two rabbits’ right knee joints, were improved if compared to group 1, (Figs. 5C, D, 7, 8). Two rabbits died in the initial phase of the study and were therefore excluded from the modified Mankin’s score evaluation. Mankin′s score in the HA plus ropivacaine hydrochloride plus triamcinolone acetonide group was 6 points, which corresponds to grade II OA. Therefore, a significant decrease in Mankin’s score was observed in this group versus group 1 (*p* < 0.01). No significant difference in Mankin’s score was observed when comparing treatment with HA alone versus HA plus ropivacaine hydrochloride plus triamcinolone acetonide.

## Discussion

The present study outlines the efficacy of a high viscoelastic divinyl sulfone cross-linked HA in a rabbit model of collagenase-induced knee OA. The rationale of using such a densely coating compound in the knee joint is to relieve the painful periosteal friction during the movement, and to help fibroblastic reaction to replace the fragmented cartilage, lubricating the synovial space and eventually promoting chondrocyte differentiation. In our study the mix of HA plus ropivacaine hydrochloride plus triamcinolone acetonide has been compared with HA alone because, on the basis of our long-standing clinical experience, especially in severely inflamed knees presenting synovial effusion, immediate pain relief, due to local anesthetic and anti-inflammatory steroid injection helps recover the joint function soon after infiltration. In our clinical experience, the addiction of highly cross-linked HA is helpful to counteract inflammation and take control of further cartilage breakdown in the short- to medium-term follow up.

Under a clinical perspective, an instant physical mix of triamcinolone acetonide and HA, immediately before IA injection and contemporary injection of ropivacaine hydrochloride or other suitable local anesthetics, may give a better functional and morphological restoration.

In the present experimental study, however, no substantial advantage has been achieved respect to HA alone. This result is not in agreement with previous findings from Karakurum et al. (2003) where a combination of HA and cortisone resulted more effective in treating experimental OA-related cartilage degeneration if compared to HA alone. An explanation for these results may be the difference in the animal model used by Karakurum and colleagues, i.e. a model created by resection of the cruciate ligament. Furthermore, they used a non-cross-linked HA and a higher dose of cortisone (15 mg/ml).

In summary, even if the spontaneous arthritic knee pathogenesis in humans is quite different from the rabbit’s factitious knee, the present study emphasizes the independent role of densely cross-linked glycosaminoglycan in counteracting the degenerative process, supporting, at least partially, the functional homeostasis due to its lubricating effect. However, our study presents a limitation consisting in the limited number of animals used.

## Conclusions

In the present study histopathological findings from the control group showed that IA administration of collagenase induced osteoarthritic changes including degradation of articular cartilage, synovial inflammation, tidemark destruction, remodelling of subchondral bone structure, osteophyte-like structure formation and excavation in the knee joint. In treated animals, some degenerative changes included superficial fibrillation of articular cartilage, vertical fissures extending into the deep zones and chondrocyte degeneration. These changes give evidence on the reparative process of articular cartilage. This process included complex chondrons, fibrocartilage tissue regeneration within denuded articular surface in the superficial zone, and new vessel formation (angiogenesis) which resulted in discontinuity of chondral zone and subchondral bone trabeculae as well as proliferation of fibrous tissue formation that were prominent at the margin of articular surface and also in the superficial zone. In treatment group animals, normal articular cartilage was not observed and there was a newly repaired fibrocartilaginous or cartilage-like tissue. A moderate loss of Safranin-O staining in right knee joint hyaline cartilage matrix was observed in control group, while a severe loss of Safranin-O staining was observed in treatment groups. Loss of matrix staining can be attributed to transient endogenous proteoglycan degradation or to proteoglycan diffusion into fixative during storage in formalin. Histological analysis of sections of right knee joints at lesion sites showed a significant decrease in Mankin’s score in groups treated with HA alone or in combination with ropivacaine hydrochloride and triamcinolone acetonide versus control. This evidence was consistent with strong articular degenerative changes in controls’ right knee joints (grade III OA), while the treatment groups revealed less severe articular degenerative changes (grade II OA).
